# Supplementary material for: Potential Onco-Suppressive Role of miR122 and miR144 in Uveal Melanoma through ADAM10 and C-Met Inhibition
Source: Cancers (Basel). 2020 Jun 4;12(6):1468. doi: 10.3390/cancers12061468 (PMC7352235; doi:10.3390/cancers12061468)
Supplement: Supplementary file 1 [file cancers-12-01468-s001.pdf]

*Supplementary Materials*

# Potential Onco-Suppressive Role of miR122 and miR144 in Uveal Melanoma Through ADAM10 and C-Met Inhibition

**Adriana Amaro, Michela Croce, Silvano Ferrini, Gaia Barisione, Marina Gualco, Patrizia Perri, Ulrich Pfeffer, Martine J. Jager, Sarah E. Coupland, Carlo Mosci, Gilberto Filaci, Marina Fabbi, Paola Queirolo and Rosaria Gangemi**

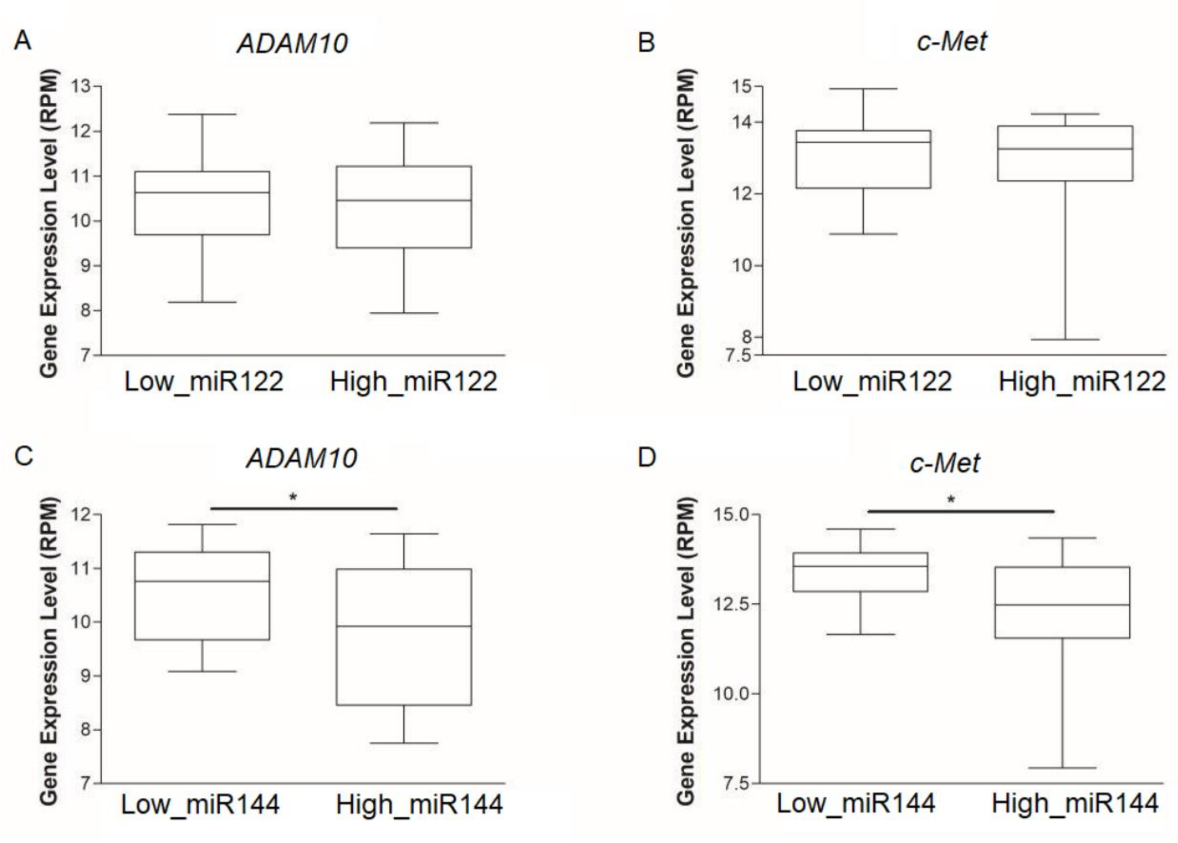


**Figure S1.** Differential expression of miR122 and 144 and correlation to *ADAM10* and *c-Met*: box and whiskers chart shows that the low expression of miR144 (bottom quartile) significantly associates with high *ADAM10* or *c-Met* expression (* $p < 0.05$). Analysis of *ADAM10* and *c-Met.* expression was performed comparing patients with high miR144 (19) with patients with low. miR144 (19), and patients with high miR122 (37) with patients with low miR122 (19).

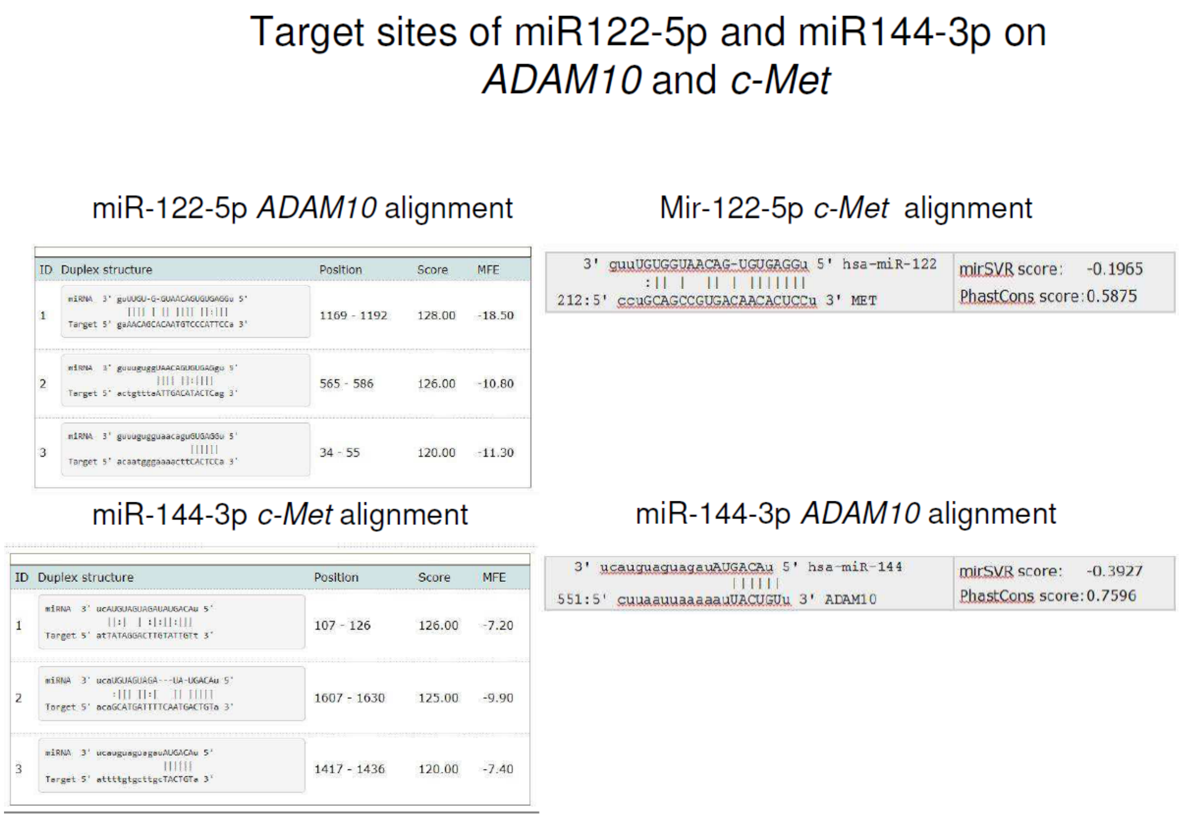


**Figure S2.** Potential target sites of miR122 and miR144 on *ADAM10* and *c-Met* by MIRANDA and miRBASE tools.

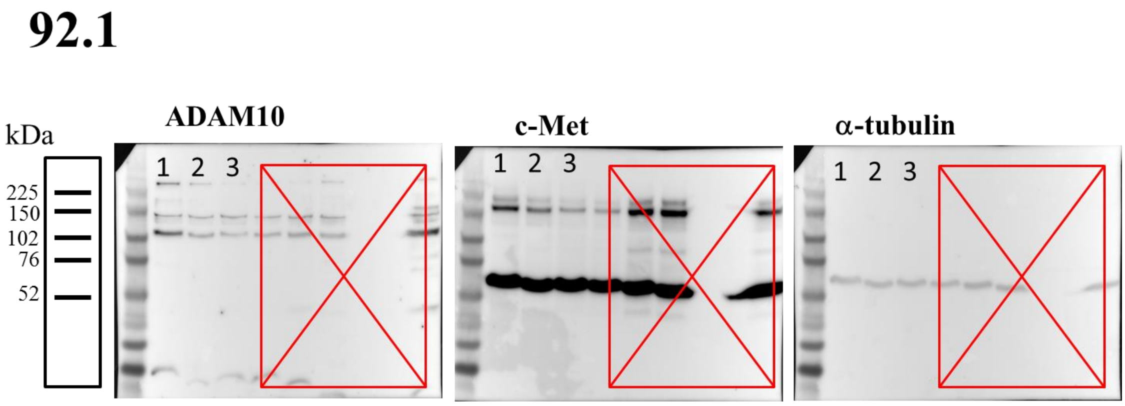


(A)

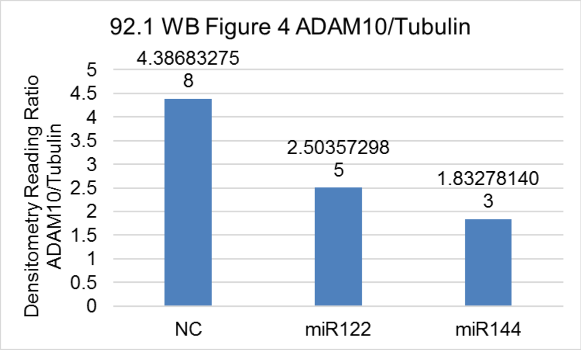


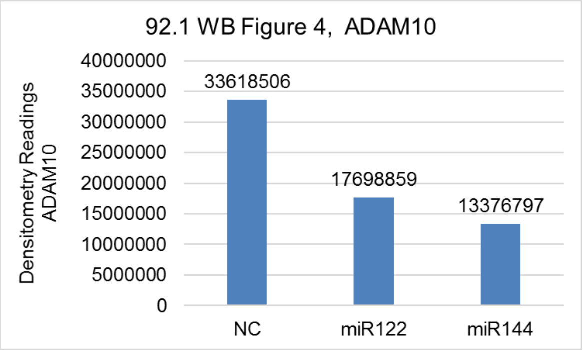

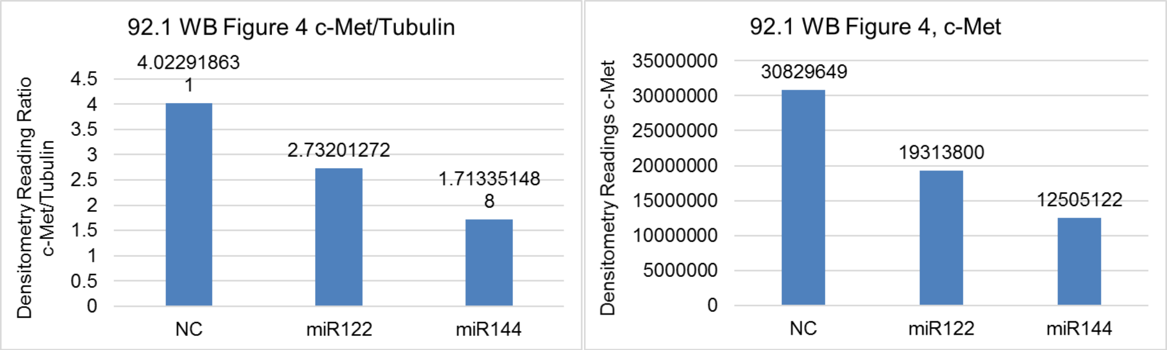


# UPMM3

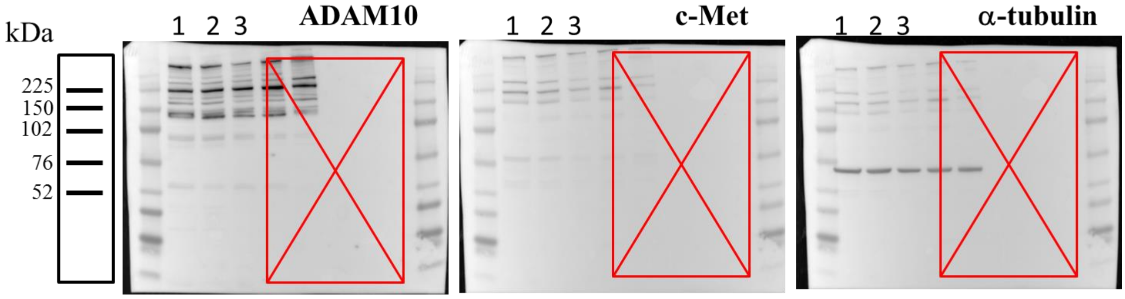


(B)

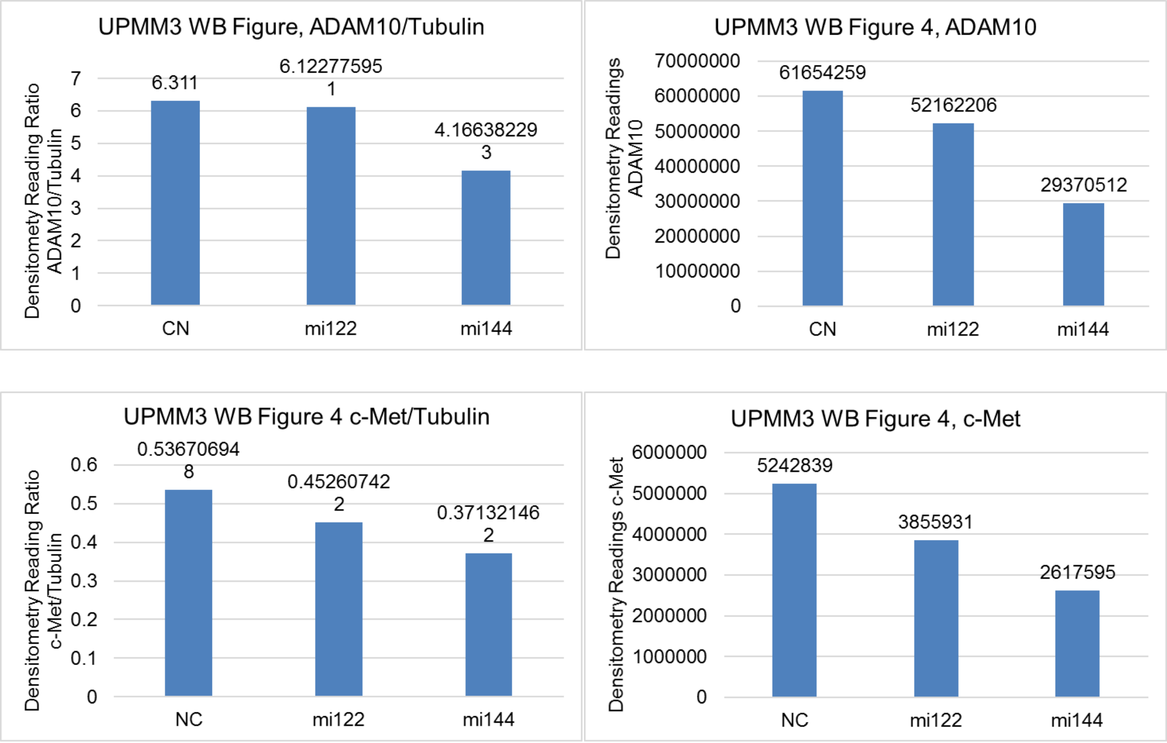


(C)

**Figure S3.** (A) Whole blots showing all the bands with molecular weight markers and densitometry presented in Figure 4. (B) 92.1 cell lysates probed with anti-ADAM10, anti-c-Met and anti- ⊚-tubulin antibodies. 1: irrelevant miRNA mimic as negative control (NC); 2: miR122; 3: miR144. (C) UPMM3

cell lysates probed with anti-ADAM10, anti-c-Met and anti- β-tubulin antibodies. 1: irrelevant miRNA mimic as negative control (NC); 2: miR122; 3: miR144.

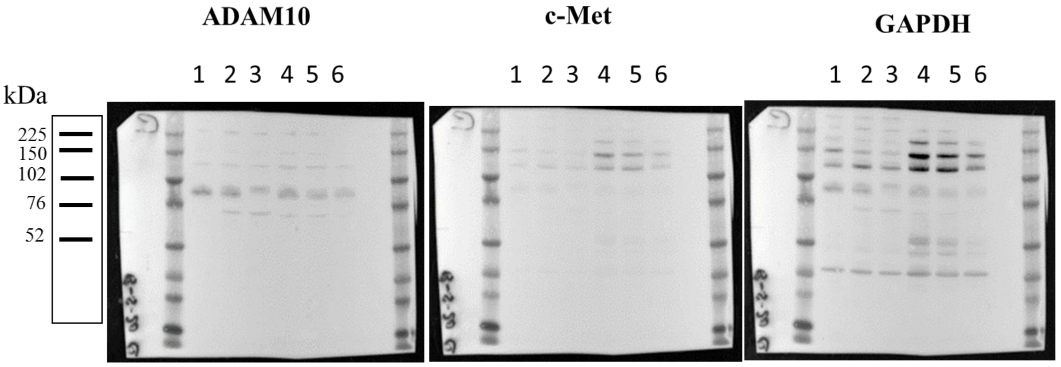


(A)

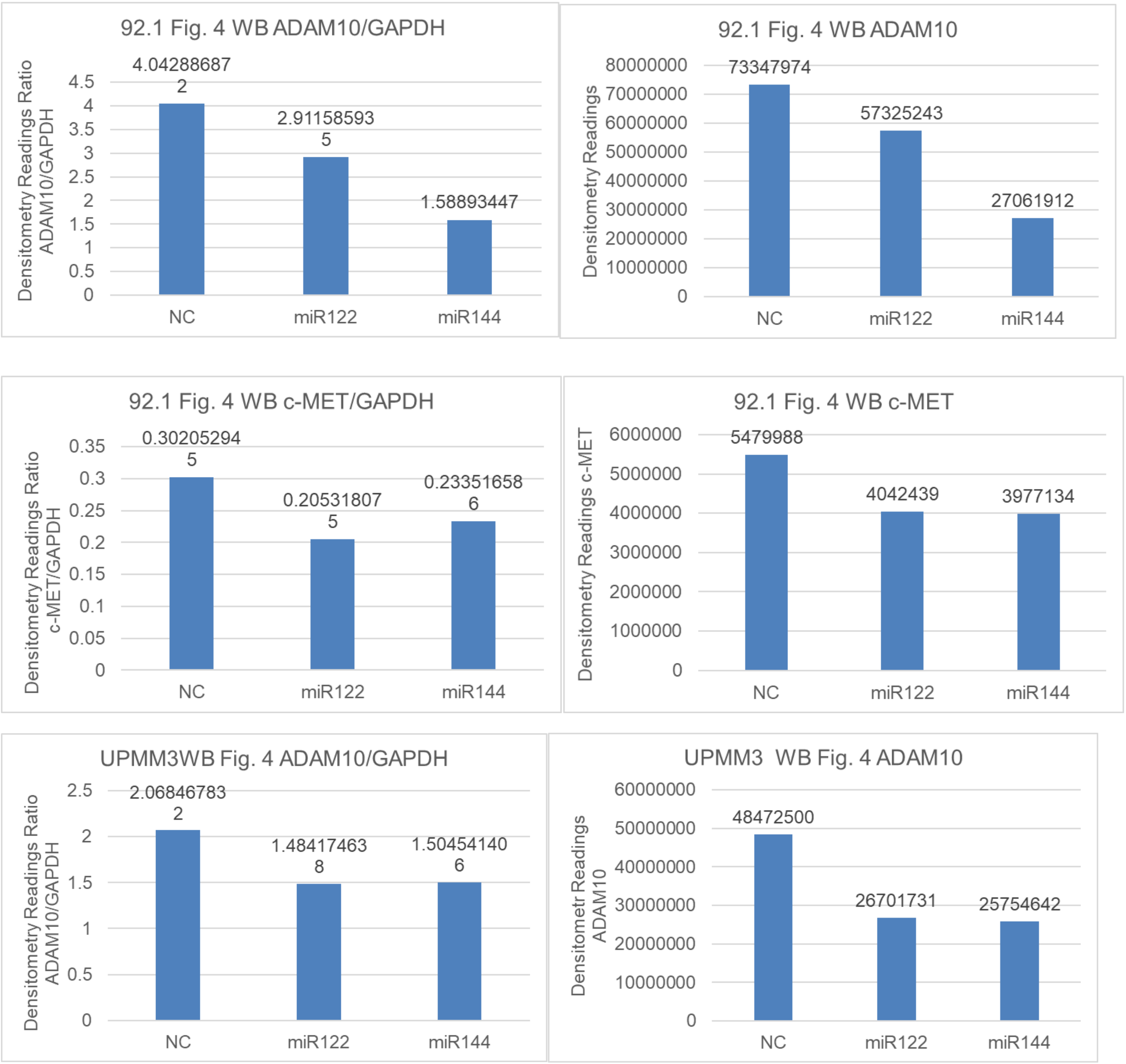

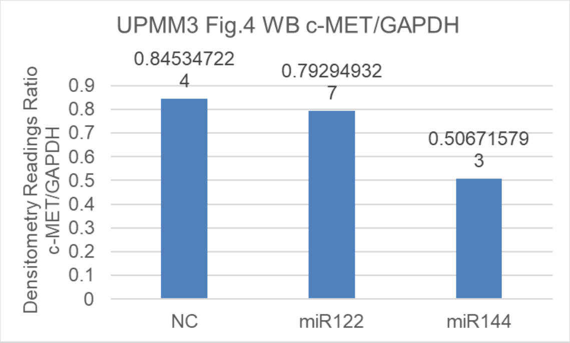
UPMM3 Fig.4 WB c-MET/GAPDH
Densitometry Readings Ratio c-MET/GAPDH
0.84534722 4
0.79294932 7
0.50671579 3
0.9
0.8
0.7
0.6
0.5
0.4
0.3
0.2
0.1
0
NC
miR122
miR144

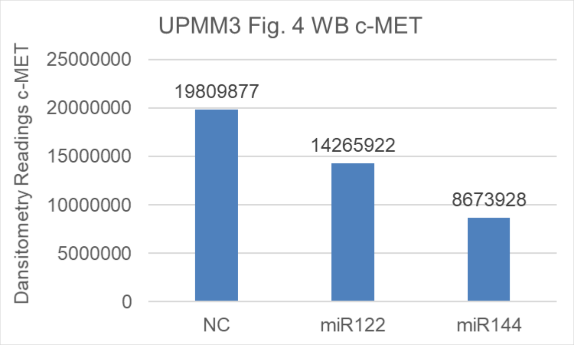
UPMM3 Fig. 4 WB c-MET
Dansitometry Readings c-MET
25000000
20000000
15000000
10000000
5000000
0
19809877
14265922
8673928
NC
miR122
miR144

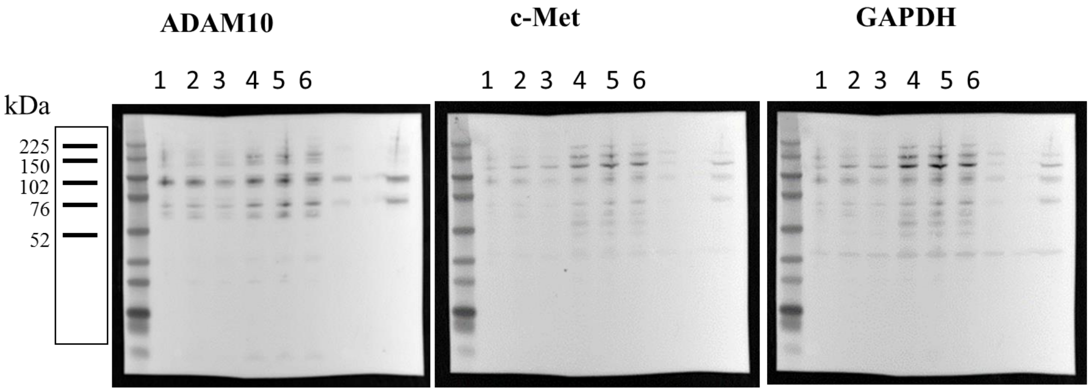
ADAM10
c-Met
GAPDH
1 2 3 4 5 6
1 2 3 4 5 6
1 2 3 4 5 6
kDa
225
150
102
76
52

(B)

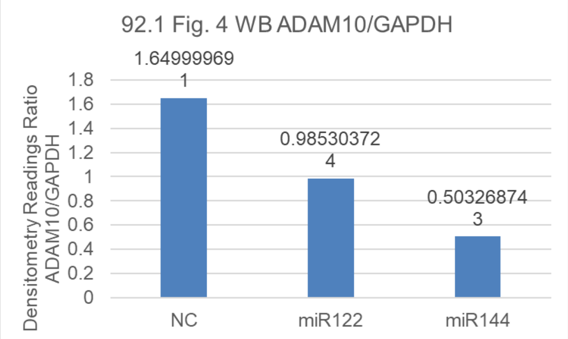
92.1 Fig. 4 WB ADAM10/GAPDH
Densitometry Readings Ratio ADAM10/GAPDH
1.64999969 1
0.98530372 4
0.50326874 3
1.8
1.6
1.4
1.2
1
0.8
0.6
0.4
0.2
0
NC
miR122
miR144

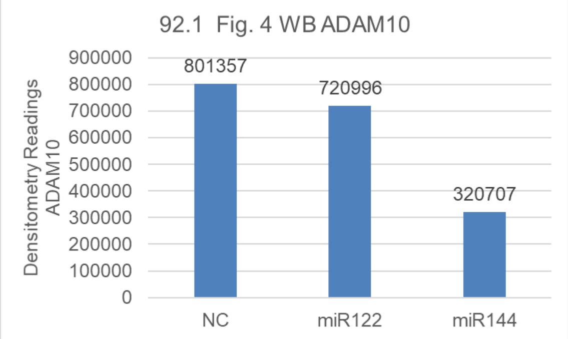
92.1 Fig. 4 WB ADAM10
Densitometry Readings ADAM10
900000
800000
700000
600000
500000
400000
300000
200000
100000
0
801357
720996
320707
NC
miR122
miR144

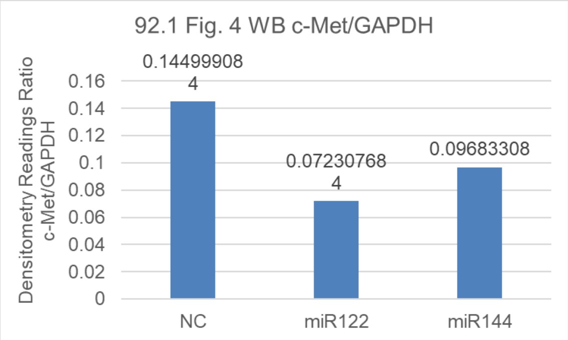
92.1 Fig. 4 WB c-Met/GAPDH
Densitometry Readings Ratio c-Met/GAPDH
0.14499908 4
0.07230768 4
0.09683308
0.16
0.14
0.12
0.1
0.08
0.06
0.04
0.02
0
NC
miR122
miR144

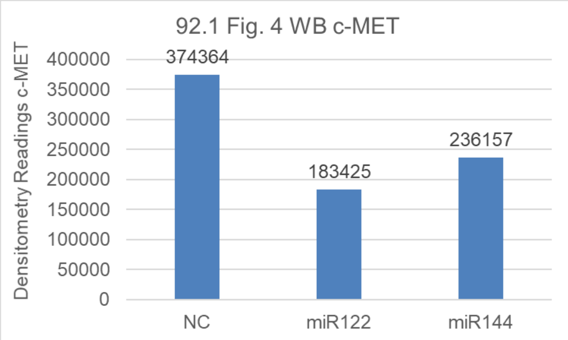
92.1 Fig. 4 WB c-MET
Densitometry Readings c-MET
400000
350000
300000
250000
200000
150000
100000
50000
0
374364
183425
236157
NC
miR122
miR144

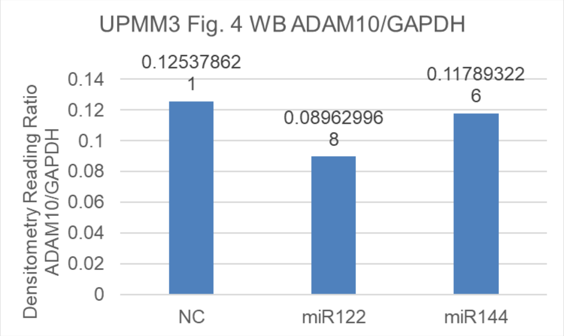
UPMM3 Fig. 4 WB ADAM10/GAPDH
Densitometry Reading Ratio ADAM10/GAPDH
0.12537862 1
0.08962996 8
0.11789322 6
0.14
0.12
0.1
0.08
0.06
0.04
0.02
0
NC
miR122
miR144

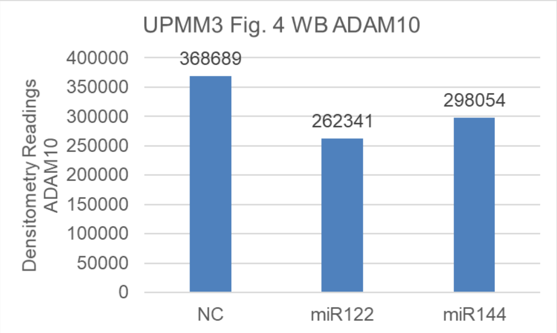
UPMM3 Fig. 4 WB ADAM10
Densitometry Readings ADAM10
400000
350000
300000
250000
200000
150000
100000
50000
0
368689
262341
298054
NC
miR122
miR144

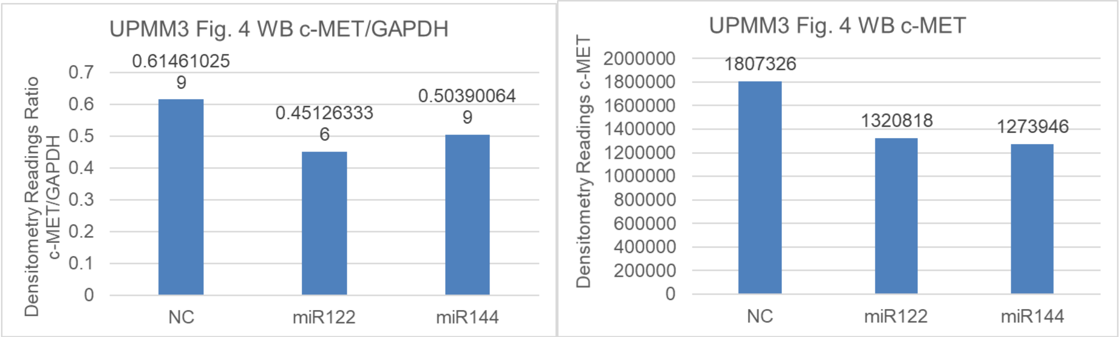


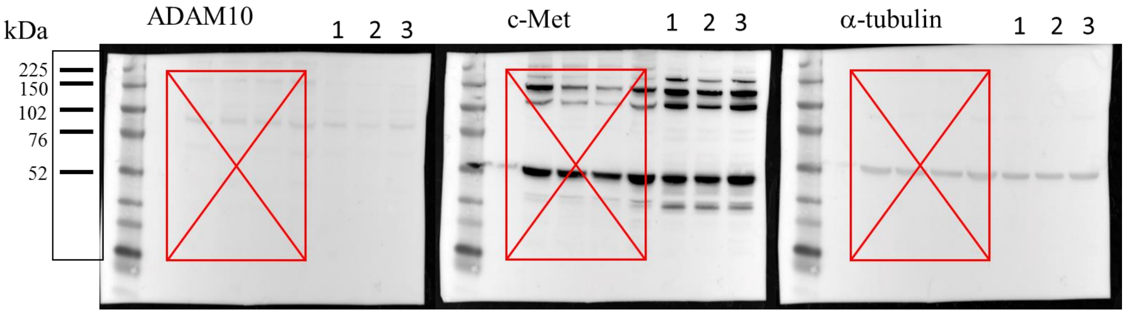


(C)

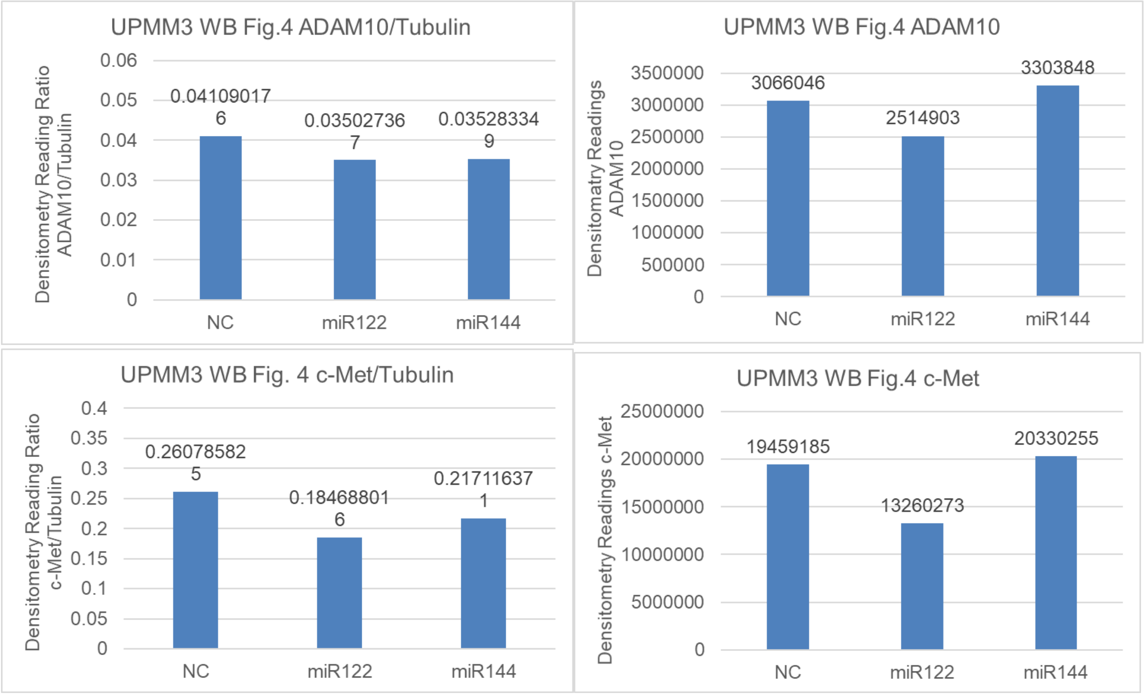


(D)

**Figure S4.** (A) Whole blots showing all the bands with molecular weight markers and relative densitometry used to obtain histogram in Figure 4 panel B. (B) 92.1 and UPMM3 cell lysates probed with anti-ADAM10, anti-c-Met and anti GAPDH antibodies. 92.1: 1: Negative Control (NC); 2: miR122; 3: miR144. UPMM3: 4: Negative Control (NC); 5: miR122; 6: miR144. (C) 92.1 and UPMM3 cell lysates probed with anti-ADAM10, anti-c-Met and anti GAPDH antibodies. 92.1: 1: Negative Control (NC); 2: miR122; 3: miR144. UPMM3: 4: Negative Control (NC); 5: miR122; 6: miR144. (D) UPMM3 cell lysates probed with anti-ADAM10, anti-c-Met and anti ⊚-tubulin antibodies. 1: Negative Control (NC); 2: miR122; 3: miR144.

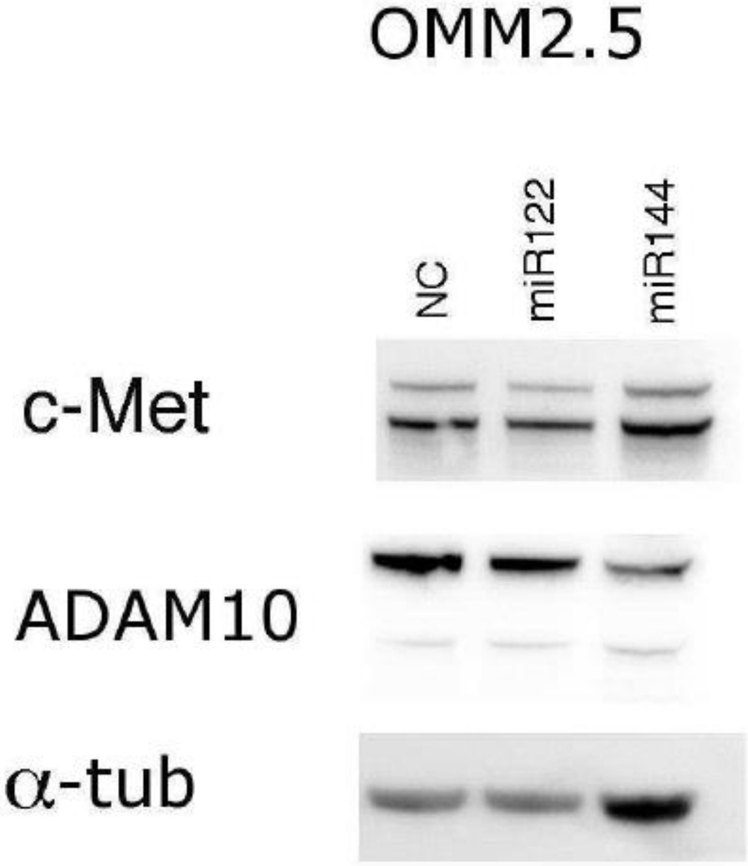


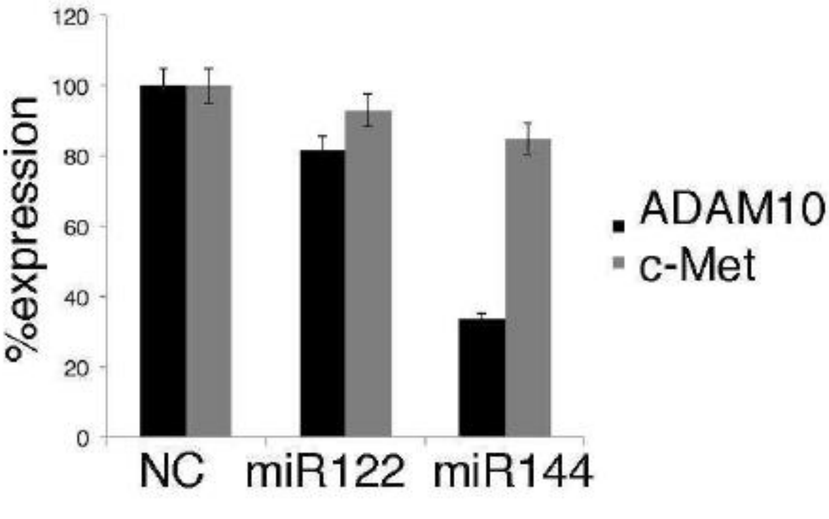


**Figure S5.** Western blot analysis of ADAM10 and c-Met expression in miR mimic transfected OMM2.5 cells: ADAM10 and c-Met proteins are down-modulated by mimic miR122. or miR144 transfection in OMM2.5 cell lines. Histograms show the quantification of band intensity as percentage expression of the irrelevant miRNA mimic, control (NC). A representative experiment of three is shown.

# OMM2.5

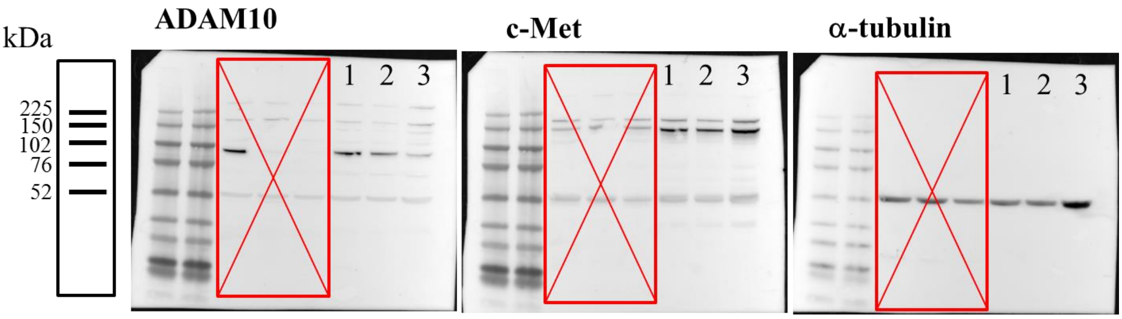


(A)

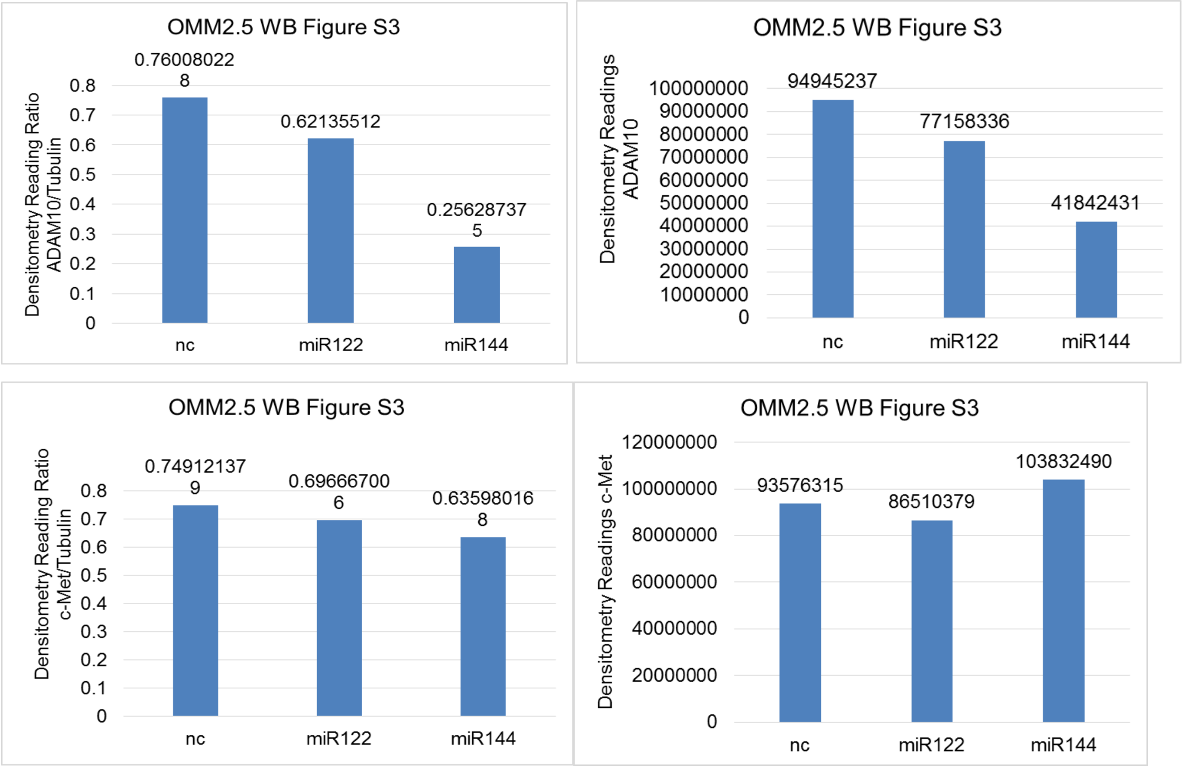


(B)

**Figure S6.** (A) Whole blots showing all the bands with molecular weight markers and relative densitometry presented in Figure S5. (B) OMM2.5 cell lysates probed with anti-ADAM10, anti-c-Met and anti- ⊚-tubulin antibodies. 1: irrelevant miRNA mimic as negative control (NC); 2: miR122; 3: miR144.

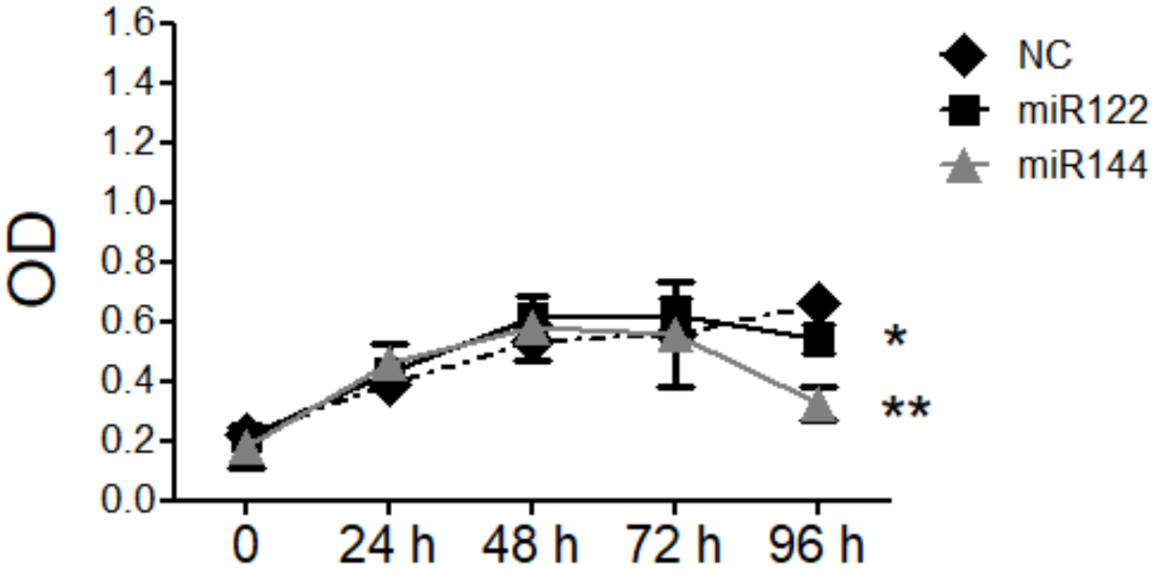


**Figure S7.** Effects of miR122 and miR144 mimic transfection on UPMM3 proliferation: miR144 and miR122 mimics transfection significantly inhibit UPMM3 cell line proliferation only 96 h after transfection, in respect to irrelevant miRNA mimic control (NC) (* $p < 0.05$; ** $p < 0.01$).

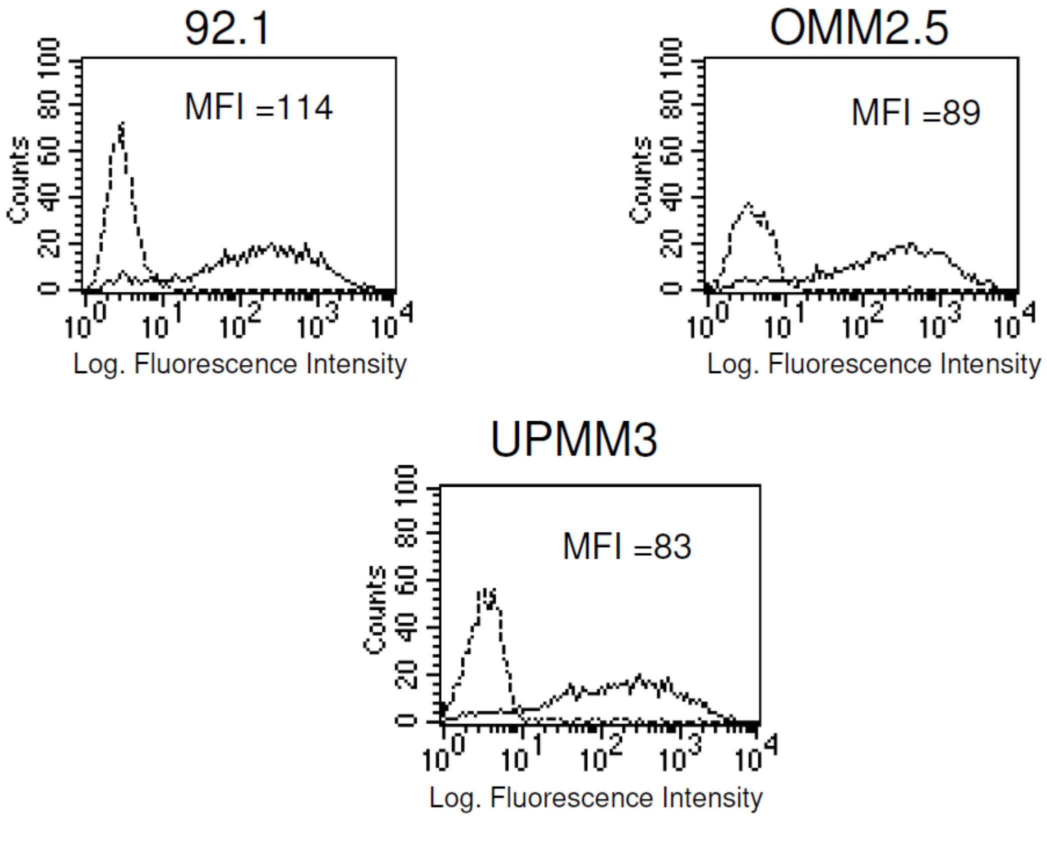


MFI= (sample Mean- CTR Mean)/CTR Mean

**Figure S8.** Histogram plots of 92.1, OMM2.5 and UPMM3 cell lines transfected with fluorescent oligonucleotides and analysed by flow cytometry: Mean Florescence intensity was. calculated by the formula: MFI = (sample Mean- CTR Mean)/CTR Mean.

© 2020 by the authors. Licensee MDPI, Basel, Switzerland. This article is an open access article distributed under the terms and conditions of the Creative Commons Attribution (CC BY) license (http://creativecommons.org/licenses/by/4.0/).
